# Genomic architecture and population structure of *Boreogadus saida* from Canadian waters

**DOI:** 10.1038/s41598-024-69782-w

**Published:** 2024-08-20

**Authors:** Trevor T. Bringloe, Audrey Bourret, David Cote, Roux Marie-Julie, Jennifer Herbig, Dominique Robert, Maxime Geoffroy, Geneviève J. Parent

**Affiliations:** 1 Fisheries and Oceans Canada, Maurice Lamontagne Institute, Mont-Joli, QC G5H 3Z4 Canada; 2 Fisheries and Oceans Canada, Northwest Atlantic Fisheries Centre, St. John’s, NL A0G 2M0 Canada; 3 Centre for Fisheries Ecosystems Research, Fisheries and Marine Institute of Memorial, University of Newfoundland, St. John’s, A1C 5R3 Canada; 4 Institut Des Sciences de La Mer, Université du Québec à Rimouski, Rimouski, QC G5L 3A1 Canada; 5 Department of Arctic and Marine Biology, UiT The Arctic University of Norway, 9036 Tromsø, Norway

**Keywords:** Arctic cod, Polar cod, Inversion, Hybridization, Introgression, Population genetics, Genome evolution

## Abstract

The polar cod, *Boreogadus saida,* is an abundant and ubiquitous forage fish and a crucial link in Arctic marine trophic dynamics. Our objective was to unravel layers of genomic structure in *B. saida* from Canadian waters, specifically screening for potential hybridization with the Arctic cod, *Arctogadus glacialis,* large chromosomal inversions, and sex-linked regions, prior to interpreting population structure. Our analysis of 53,384 SNPs in 522 individuals revealed hybridization and introgression between *A. glacialis* and *B. saida*. Subsequent population level analyses of *B. saida* using 12,305 SNPs in 511 individuals revealed three large (ca. 7.4–16.1 Mbp) chromosomal inversions, and a 2 Mbp region featuring sex-linked loci. We showcase population structuring across the Western and Eastern North American Arctic, and subarctic regions ranging from the Hudson Bay to the Canadian Atlantic maritime provinces. Genomic signal for the inferred population structure was highly aggregated into a handful of SNPs (13.8%), pointing to potentially important adaptive evolution across the Canadian range. Our study provides a high-resolution perspective on the genomic structure of *B. saida*, providing a foundation for work that could be expanded to the entire circumpolar range for the species.

## Introduction

Polar cod (*Boreogadus saida*) is an abundant and ubiquitously distributed, circumpolar gadid. It is an important consumer of a variety of zooplanktonic species^1–4^, and is itself an important food source for Arctic predators, contributing disproportionately to energy transfer to higher trophic levels in some areas^5–7^. Aggregations of *B. saida* are an important food source for narwhal (*Monodon monoceros*^8^), beluga (*Delphinapterus leucas*^9^), ringed seal (*Pusa hispida*^10^), and various marine birds such as thick-billed murres (*Uria lomvia)* and northern fulmars (*Fulmarus glacialis*^5^). *B. saida* is found throughout Arctic ice shelves (particularly earlier life stages^11,12^), where it can flourish due to the synthesis of antifreeze glycoproteins^13^. Given the central role played by *B. saida* in Arctic food webs, its polar distribution, and potential adaptation for Arctic conditions, climate change is anticipated to impact this species, precipitating profound changes to ecological services in Arctic waters^12^. Potential non-mutually exclusive threats include the introduction of new predators and competitors and shifting prey distributions through the borealization of Arctic waters, diminishing sea ice extent and thickness that serves as habitat for early life stages, warming surface waters impacting egg development and hatching success, and increased human activity in a more seasonally open Arctic ocean^12,14,15^. Robust baseline understanding of the diversity and connectivity of *B. saida* in Arctic and subarctic regions is needed to better anticipate the impacts of a changing climate and anthropogenic stressors on the species and marine communities.

Early genetic studies in *B. saida* showed weak population structure and high connectivity across global populations. Among the earliest molecular perspectives, Fevolden et al.^16^ found no evidence for differentiation between populations of Greenland (Disko Bay and Denmark Strait), Svalbard, and Northern Norway using the nuclear locus *Syp*I and 38 randomly amplified polymorphic nuclear loci, a result further confirmed by Pálsson et al.^17^ using mitochondrial markers. In more recent work, Maes et al.^18^ also confirmed no population structure between Svalbard and the Eurasian basin using nine microsatellites, which the authors attribute to high gene flow between study regions. Quintela et al.^19^ found similar results using 116 single nucleotide polymorphic (SNP) loci, but did detect isolation by distance between the East Siberian and Laptev Seas, and the Eurasian Basin locations. Low levels to complete absence of population structure were further reported in the Chukchi Sea and adjacent Beaufort Sea populations using mitochondrial *cyt*b and microsatellites^20,21^), and in Russian Arctic seas using microsatellites^22^. In contrast, differentiation between fjord-dwelling and shelf-dwelling *B. saida* populations have been reported from the East Atlantic using microsatellites^23^, suggesting local divergence could play a primary role in driving genetic structure in *B. saida*. Lending credence to this hypothesis, Hill et al.^24^ demonstrated that fjord and offshore dwelling specimens differentially expressed approximately 2000 genes. In contrast to these works, a global-scale study of *B. saida* populations using nine microsatellites was published by Nelson et al.^25^, who revealed four populations corresponding to (1) the East Canadian Arctic, (2) the West Canadian Arctic (i.e. East Beaufort Sea), (3) the US Arctic Seas (Bering, Chukchi and West Beaufort Seas), and (4) the European Arctic Seas (Greenland, Iceland, and Laptev Sea). Besides demonstrating that population structure did exist at global scales, the authors also noted our understanding of *B. saida* population structure would benefit from higher density marker datasets, which can be provided by genomics.

Interpreting genomic structure in *B. saida* is likely to come with specific challenges. Among the complications with resolving population structure in *B. saida* are potential hybridizations. These events can have profound evolutionary consequences, ranging from adding new opportunities for adaptive evolution to eroding the integrity of species barriers, resulting in loss of biodiversity^26,27^. In gadids, extensive introgression potentially facilitated early evolution in some species, as evidenced by non-random sharing of alleles between walleye pollock (*Gadus chalcogrammus*), Pacific cod (*Gadus macrocephalus*), and Greenland cod (*Gadus ogac*)^28^ (as noted by the authors Pacific cod and Greenland cod may represent the same species^29^). More relevant for population level analyses are known contemporary hybridization between *B. saida* and the Arctic cod *Arctogadus glacialis*, which overlaps with *B. saida* in the Canadian Arctic^30^ and has similar ice-associated habitat^31^. At least one hybrid individual has been confirmed with molecular data by Wilson et al.^32^. Identifying *B. saida* and removing individuals with introgressed *A. glacialis* information are important steps to ensure species level information is not conflated with population level dynamics, and for better understanding the evolutionary trajectory of both species. In addition to hybridizations, the genomic architecture of Canadian *B. saida* is likely to feature large inverted chromosomal segments as reported for gadid species in general^33^, which results in reduced recombination between inverted genotypes that creates highly differentiated regions between genomes. This differentiation can subsequently offer important genomic variability on which adaptive evolution can act. These inversions are hypothesized to provide a template for adaptive divergence in cod populations, a mechanism explored in the Atlantic cod, *Gadus morhua*^34,35^. By mapping genomic data for *B. saida* to the reference genome of *G. morhua*, Einarsson et al.^36^ suggested the presence of inversions on chromosomes 1 and 7.

In this study, we describe the genomic structure of *B. saida* over wide latitudinal and longitudinal gradients covering Canadian Arctic and subarctic regions (from 48.25° to 76.39° N, and 53.71° to 138.65° W). Given the complexities detailed above, we proceeded in a stepwise fashion, starting with the objective to determine whether *B. saida*/*A. glacialis* hybrids were present in our dataset, and if so, the extent and distribution of introgressed information. Second, we screened our data for highly differentiated regions of the genome, in particular sex-linked regions and inverted genomic blocks acting as potential supergenes. Third, and once the aforementioned factors were removed from our dataset, we investigated patterns of population structuring for *B. saida* in Canadian waters. Our expectation, based on available genomic evidence for Gadidae^32–36^, was that the genomic structure of *B. saida* would be layered and complex. Our analysis of 1000 s of SNPs provided further insights related to introgression events between *B. saida* and *A. glacialis*, large inverted genomic blocks similar to *G. morhua*, sex-linked loci of the genome, and new details about population structure across Canadian Arctic and subarctic waters.

## Results

**Figure 1:**
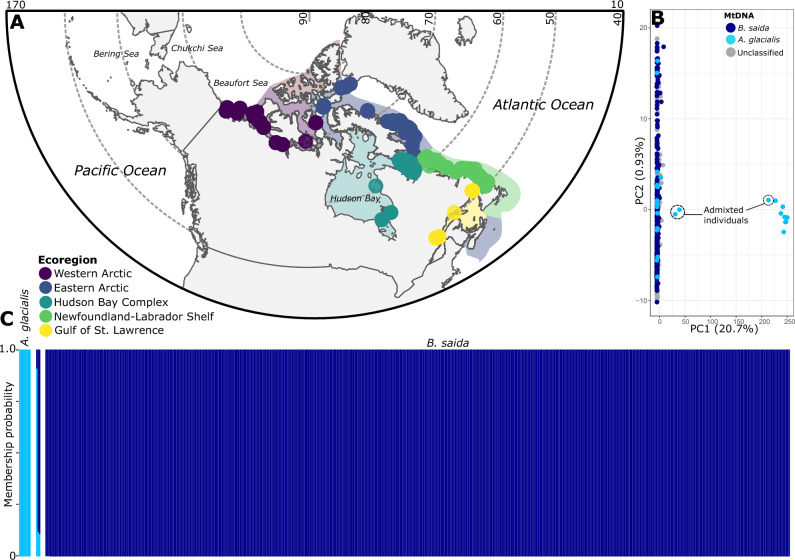
(**A**) Sampling locations for *Boreogadus saida* and *Arctogadus glacialis* specimens considered in this study. Specimens were sampled across five marine ecoregions in Canadian Arctic and subarctic waters^73^. (**B**) Principal Component Analysis of 53,384 SNPs in 522 *B. saida* and *A. glacialis* individuals, with some *B. saida* individuals (left) harboring *A. glacialis* (right) MtDNA (admixed individuals noted); note, dots overlap, and some individuals were unable to be classified with mitochondrial SNPs. (**C**) ADMIXTURE analysis of the same 522 individuals. Map polygons were sourced from *rnarturalearth* R package^98^ and set to polar projection.

To infer the genomic structure of *B. saida*, we analysed 522 specimens from 92 sampling sites from the Western Arctic to the Gulf of St. Lawrence marine ecoregions (Fig. 1A). The initial analysis of 53,384 SNPs indicated the presence of nine highly differentiated individuals that were confirmed as *A. glacialis* based on mitochondrial DNA SNPs (Fig. 1B). Eight of these individuals were sampled in the Western Arctic while the remaining one originated from the Eastern Arctic (Table 1). Another 19 individuals belonging to a larger cluster identified as *B. saida* also had *A. glacialis* mitochondrial DNA (Fig. 1B). Individuals with *B. saida* nuclear DNA and *A. glacialis* mitochondrial DNA were sampled from all marine ecoregions investigated except for the Hudson Bay Complex, with the highest proportions in the Western Arctic (Table 1). Three individuals exhibited admixed proportions between *B. saida* and the *A. glacialis* bearing an ~ 11% membership probability in favour of the other species cluster (Fig. 1C). Such admixed individuals were collected in the Western Arctic ecoregion (Table 1).

**Table 1:** Percentage of specimens from Canadian ecoregions that were *Arctogadus glacialis*, putative introgressed between *A. glacialis* and *Boreogadus saida*, and *B. saida* with *A. glacialis* mitochondrial DNA. Sample sizes are provided in brackets.

Ecoregion	Sample size	Percentage of *A. glacialis*	Percentage of introgressed (*A. glacialis* x *B. saida)*	Percentage of *B. saida* with *A. glacialis* mtDNA
Western Arctic	117	6 (7)	2.5 (3)	7.7 (9)
Eastern Arctic	168	0.6 (1)	0	1.8 (3)
Hudson Bay Complex	58	0	0	0
Newfoundland-Labrador Shelf	118	0	0	2.5 (3)
Gulf of St. Lawrence	61	0	0	3.3 (2)

**Figure 2:**
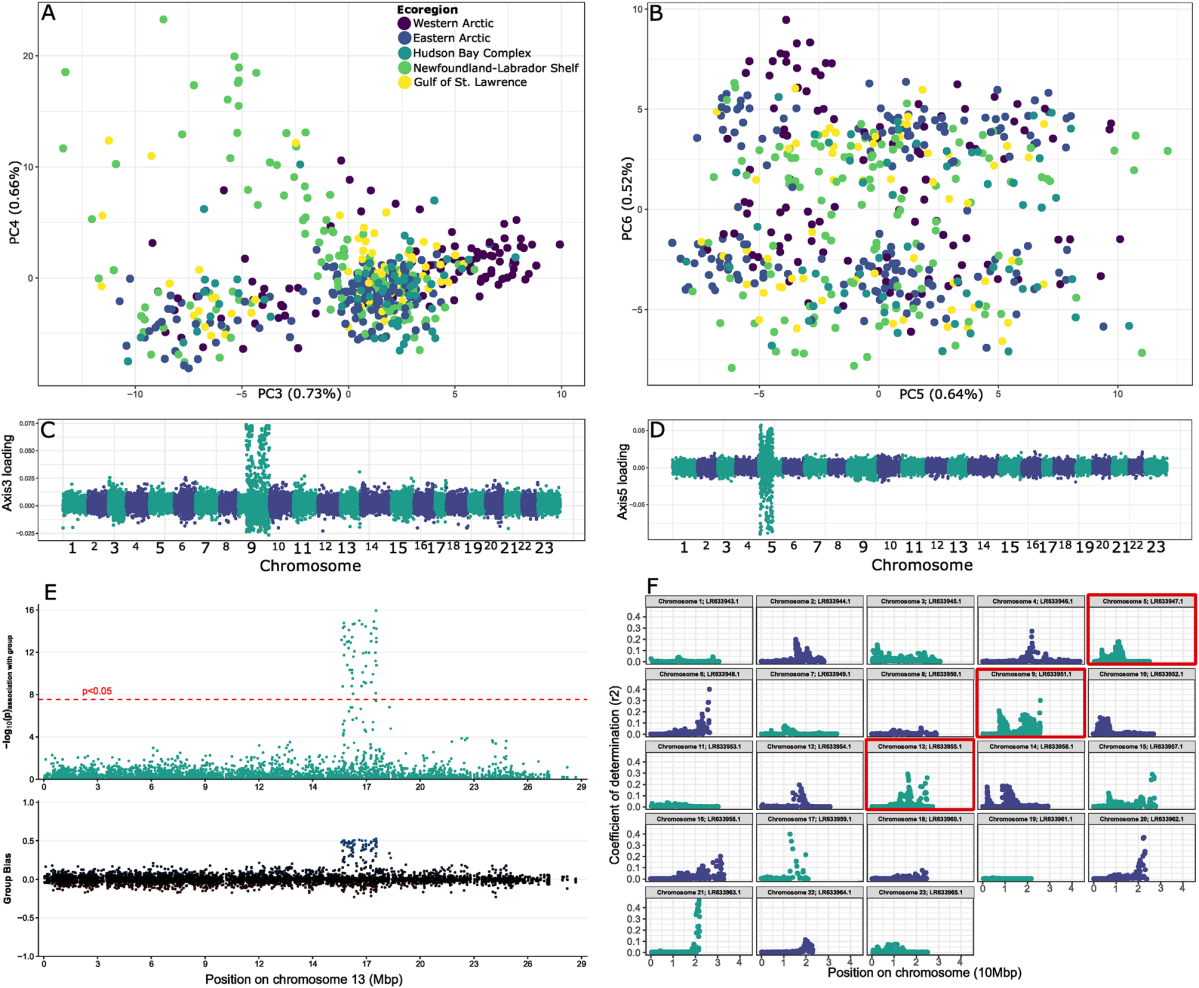
Initial population results of Canadian *Boreogadus saida* based on genome-wide SNPs (n = 12,305 SNPs). (**A**) Principal Component Analysis (PCA) axes 3 and 4 demonstrating lack of geographic specificity to clustering (PCs 1 and 2 depict structure and allele loadings in Fig. 3). (**B**) PCA axes 5 and 6 demonstrating similar results. Allele loading profiles (**C** and **D**) linked to chromosomes 9 (LR633951.1) and 5 (LR633955.1), respectively. (**E**) RADSex results showing sex bias on chromosome 13 (note x-axis indicates beginning position of mapped reads within the chromosome). Upper panel presents the probability of association with sex, with the dashed line indicating a *p*-value of 0.05 after Bonferroni correction, while the lower panel indicates the direction of bias, where positive values indicate the bias toward males and negative, toward females. (**F**) Linkage disequilibrium (LD) averaged for all loci pairs separated by 100 kbp-5 Mbp. Spikes in LD indicate sites are correlated with adjacent sites to within 5 Mbp, suggesting these regions are inherited as a unit. Chromosomes 5, 9, and 13 are highlighted by red boxes, but note LD spikes are present on other chromosomes.

A subsequent set of analyses to detect chromosomal inversions and including SNPs from only non-hybrid *B. saida* individuals (n = 511, SNPs = 12,305) revealed clustering patterns lacking any obvious geographical structure among the ecoregions considered. These patterns included two widespread clusters on PCA axis 3, and a further two clusters on axis 6 (Fig. 2A,B). Axis loadings also revealed highly aggregated signals on chromosomes 9 and 5, respectively (Fig. 2C,D). The loadings on chromosome 9 featured two notable peaks between positions 7,350,621–11,315,580 and 17,597,431–23,417,670 (Fig. 2C). A third genomic region was also identified as biased towards sex, though no geographic associations were found. A significant departure from random sex association was detected on a segment of chromosome 13 at positions 16,060,190–17,944,656 Mbp, which was biased toward males (heterogametic sex, Fig. 2E). The aggregated signals on the three chromosomes were further corroborated with linkage disequilibrium measurements (Fig. 2F). Other notable peaks of linkage disequilibrium were also present on other chromosomes (Fig. 2F). A PCA of SNPs located within the putative inverted segments of chromosome 5 and 9 both featured three clusters (Fig. S2): two clusters with low heterozygosity, i.e., homozygous genotypes for the ancestral or the inverted segment, on each extremity of the first axis, and a third cluster with high heterozygosity, i.e., genotype heterozygous with an ancestral and inverted segment, close to the middle of the first axis (Fig. S2). All inversion genotypes lacked geographic specificity. Despite investigating 428, 634, and 680 Ensembl gene codes for the biased regions on chromosomes 5, 9, and 13, respectively, g:Profiler only revealed a few instances of potential gene enrichment for ribosomal functions and RNA processing (Fig. S3).

**Figure 3:**
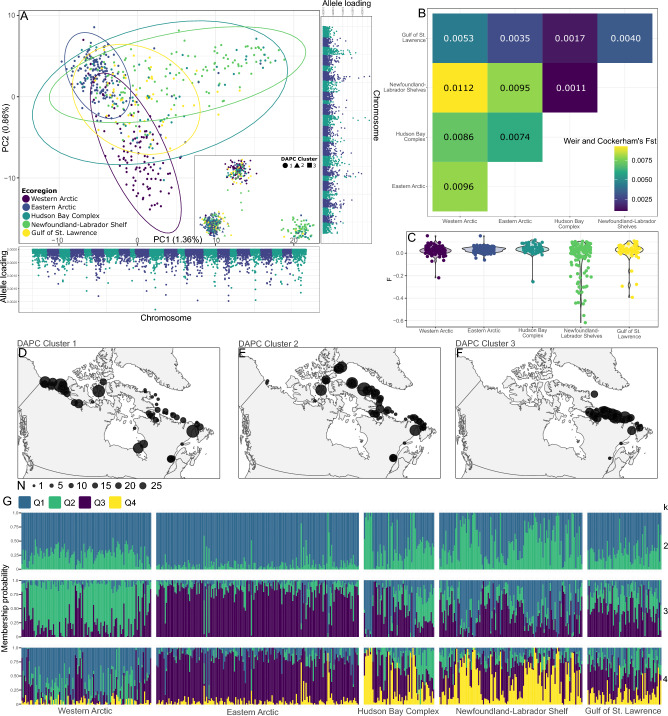
Population structure of Canadian *Boreogadus saida* (n = 11,233 SNPs). Results include: (**A**) Principal Component Analysis with Discriminant Analysis of Principal Components (DAPC) inset and discriminant analysis allele loading profiles depicted for each axis (assuming 3 clusters and ca. 80% of variation retained during analysis); (**B**) Weir and Cockerham’s FST pairwise measurements for each ecoregion (all pairwise estimations were significantly different from 0; all *p* < 0.001); (**C**) Inbreeding coefficients; geographic distribution of DAPC clusters 1–3 (**D**–**F**, respectively), where N refers to number of individual specimens genotyped; and (**G**) ADMIXTURE results at k = 2–4 (k = 3 was best supported based on cross-validation error, Fig. S1).

The analysis of population structure in *B. saida* without SNPs from the biased regions of chromosomes 5, 9, and 13 (N = 11,233 SNPs) revealed three main clusters in the study area. The PCA revealed that the majority of individuals from the Hudson Bay Complex, Newfoundland-Labrador Shelves, and some individuals from the Gulf of St. Lawrence, differed from individuals of the Western and Eastern Arctic on the first axis. Specimens from the Western and Eastern Arctic ecoregions were differentiated on PCA axis 2 (Fig. 3A), though there was considerable overlap. This clustering pattern was replicated with Discriminant Analysis of Principal Components (DAPC), and allele loadings were aggregated to some extent across most chromosomes (Fig. 3A). In line with these results, while all pairwise FST comparisons were significantly different from 0 (all *p* < 0.001), the pairwise comparison of Western Arctic and Newfoundland and Labrador Shelves ecoregions was the most differentiated (FST = 0.0112), followed by comparably high differentiation between the Western and Eastern Arctic (FST = 0.0096), between the Western Arctic and Hudson Bay complex (FST = 0.0086), and between the Eastern Arctic and the Newfoundland and Labrador Shelves (FST = 0.0095; Fig. 3B). The Gulf of St. Lawrence was the least differentiated ecoregion across all comparisons, while the Hudson Bay Complex and Newfoundland and Labrador Shelves similarly showed low differentiation (Fig. 3B). Inbreeding coefficients indicated heterozygosity was significantly different across ecoregions (*p* = 0.002). Specifically, heterozygosity appeared to be highest in one of the subarctic ecoregions, Newfoundland and Labrador Shelves. Dunn’s post hoc test confirmed *F* was significantly less in Newfoundland and Labrador Shelves compared to Hudson Bay Complex (*p* < 0.001; Fig. 3C), but did not confirm significant differences in other pairwise comparisons. Though all DAPC clusters were distributed throughout the study region, geographic patterns were still notable. Most Western Arctic specimens were associated with cluster 1 (Fig. 3D), while most specimens from the Eastern Arctic were associated with cluster 2 (Fig. 3E). The third cluster was only present in the eastern Canada distribution with a majority of samples collected along the Labrador coast (Fig. 3F). These three clusters were likely not completely reproductively isolated since higher levels of heterozygosity appeared to aggregate at the facing margins of each cluster (Fig. S4). ADMIXTURE corroborated the above structure inferred from PCA and FST, with distinct structure corresponding to the Western Arctic, the Eastern Arctic, and the continuum from Hudson Bay Complex through to the Gulf of St. Lawrence (Fig. 3G).

**Figure 4:**
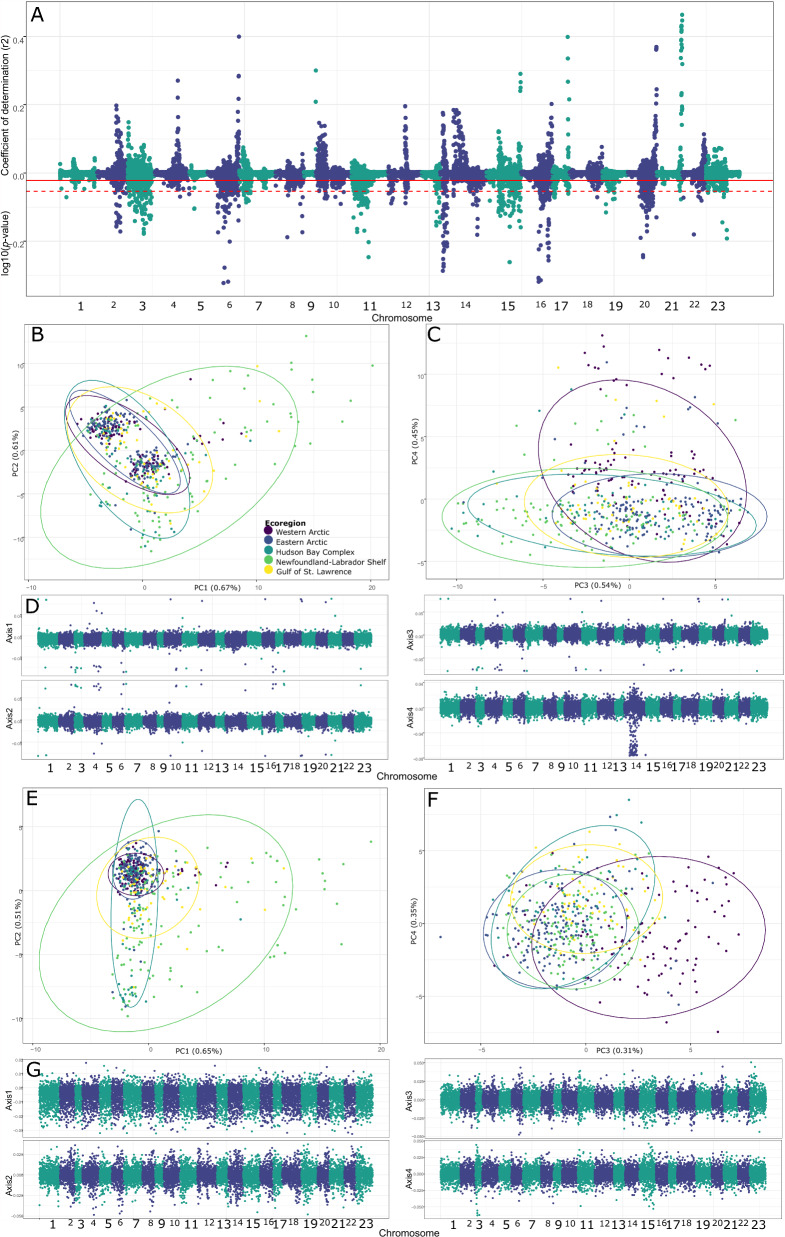
Population structure of outlier and neutral loci identified by PCAdapt in Canadian *Boreogadus saida*. (**A**) Manhattan plot of linkage disequilibrium averaged for all loci pairs separated by 100 kbp-5 Mbp (positive values) and PCAdapt log10 (*p*-values, negative values) for all loci. The solid red line depicts a threshold for candidate loci under selection based on q-values assuming a false discovery rate of 5%, whereas the dashed red line is a more conservative Bonferroni corrected threshold. (**B**) Principal Component Analyse (PCA) axes 1 and 2 based on neutral loci identified using the less conservative q-value threshold, along with (**C**) axes 3 and 4 (N = 9680 SNPs). (**D**) Allele loadings of neutral loci after filtering for linkage disequilibrium. PCA of unlinked loci (n = 7133 SNPs), depicting axes 1–2 (**E**) and 3–4 (**F**). (**G**) Allele loadings of the PCAs based on unlinked loci.

Allele loadings were notably aggregated in the above analyses (Fig. 3A), so outlier analyses were used to corroborate these results. Outlier loci contributing excessively to observed patterns in population structure were identified using both PCAdapt (Fig. 4) and BayeScan. Assuming a 5% false discovery rate, 13.8% of loci were identified as outliers with PCAdapt (n = 1553; Fig. 4A) and 4.1% with BayeScan (n = 465), with 4.0% (n = 443) identified with both approaches. Using a more conservative Bonferroni corrected threshold, 4.3% (n = 487) of loci were identified as outliers, with 71.3% (n = 347) in common with BayeScan. Loci were aggregated within chromosomes (Fig. 4A) and mirrored peaks in linkage disequilibrium (Fig. 2F). The outlier loci identified with PCAdapt replicated the population structure of the full dataset using both conservative and normal thresholds (Fig. S5). The PCA of the putative neutral loci (i.e., not identified as outlier by PCAdapt, n = 9680) identified clusters typical of inversion genotypes in PC axes 1 and 2 (Fig. 4B) and supported the inferred population structure depicted in Fig. 3 for PC axes 3 and 4 (Fig. 4C). The allele loadings indicated the signal remained aggregated in a few genomic positions, except for chromosome 14 between positions 8,874,942–16,337,747 (Fig. 4D). A PCA of the chromosome 14 biased region revealed a clustering pattern related to levels of heterozygosity or inversion genotypes (Fig. S2). The final dataset with the biased region of chromosome 14 removed, and retaining 1 SNP/inter- and intra-chromosomal linkage group (n = 7133) again reflected to a weaker extent the previously inferred population structure, with Newfoundland-Labrador Shelves and Hudson Bay Complex individuals differentiated on PCA axes 1 and 2 (Fig. 4E), and Western Arctic individuals differentiated on axis 3 (Fig. 4F). Allele loadings confirmed the aggregated signal was largely removed (Fig. 4G). Results for population analyses were nearly identical for the unlinked loci (Fig. S6), except clusters were less well defined, and only a single cluster was supported by DAPC and ADMIXTURE (Fig. S1).

## Discussion

Previous genetic studies relying on mitochondrial DNA, microsatellites, and small-scale SNP datasets (100+) have suggested population structure in polar cod, *Boreogadus saida,* is generally absent or weak owing to high connectivity across its range, though global scale structure has been inferred in some cases^19,25^. Higher resolution datasets promise to reveal genomic structure potentially missed in previous analyses, but many complications owing to the biology of gadids, such as putative chromosomal inversions, must be carefully ruled out before interpreting particular factors affecting structure such as gene flow. Using 1000s of SNPs for higher resolution, our objective was to peel back layers of genomic structure in *B. saida* from five Canadian Arctic and subarctic ecoregions, specifically screening for potential hybridizations with *Arctogadus glacialis,* and putative large genomic inversions and sex-linked regions, prior to interpreting population structure. Our analysis of 53,384 SNPs confirmed introgression of genomic information between *B. saida* and *A. glacialis*, suggesting hybrids backcross with both parental species. We also unravelled important genomic architecture suggesting inverted genomic segments on chromosomes 5, 9, and 14, with another segment linked to sex differences on chromosome 13. We found that population structure in Canadian waters is driven by a small subset of outlier loci aggregated in numerous regions throughout the genome, which are potentially the result of adaptive divergence across the North American Arctic. Once all confounding factors were removed from the SNP dataset, population structure was weak but reaffirmed patterns observed prior to removing putative inversions and adaptive loci. Our study provides new insights into the biology of *B. saida*, and relevant genomic context for protecting this species and its associated ecological functions under a changing climate.

### Interbreeding of *Boreogadussaida* and *Arctogadusglacialis*

*B. saida* co-occurs with another gadid *A. glacialis* in the Canadian Arctic. *A. glacialis* is less widely distributed and abundant, but does overlap with *B. saida* on Arctic continental shelves, mainly in the Northern Chukchi Sea, Northwest reaches of the Canadian Arctic Archipelago, Northern Greenland, and Arctic Russian waters^30,37^. The two species can be distinguished morphologically based on total gill rakers on the anterior arch, and the number of rays of the second dorsal fin and first anal fin^30^. Mitochondrial DNA^38^ and a single microsatellite marker (Gmo8) have been previously reported to distinguish the two species^39^, though as explored below, this mitochondrial DNA signal may not be an entirely reliable diagnostic criterion.

Despite differences in ecology, morphology, and genetics^7,30,39^, we found evidence of introgression between *B. saida* and *A. glacialis* based on principal component and ADMIXTURE analyses. It was possible to discriminate these admixed individuals since both parental species were genotyped in our dataset. The initial results identified nine highly differentiated individuals caught in the Western and Eastern Arctic ecoregions that were identified as *A. glacialis* on the basis of nuclear loci and mitochondrial haplotypes (Fig. 1A,B). A further 19 individuals clustering with *B. saida* for nuclear loci were genotyped for *A. glacialis* for mitochondrial haplotypes, suggesting directional introgression of *A. glacialis* mitochondrial DNA into *B. saida*. While this observation could stem from shared ancestral polymorphisms, the presence of three admixed individuals strongly suggests these patterns are due to introgression of mitochondrial DNA. Introgression of cytoplasmic DNA is not uncommon in lineages with the potential to interbreed, and has been widely reported in animals and plants undergoing range expansions^40^. If the patterns reported here are due to introgression, this was potentially initiated during recolonization of the Arctic following the last glacial maximum ca. 12 ka^41^ once overlapping species ranges were established, though time calibrated analyses are needed before this can be confirmed. Given both species spawn under sea ice, with sympatric development of eggs and larvae^31^, the potential for interbreeding appears biologically highly plausible. Because organellar genomes are uniparentally inherited, the *A. glacialis* haplotype has potentially become a divergent sequence in the landscape of mitochondrial genetics of *B. saida*. As such, it is imperative that the identification of specimens does not rely on mitochondrial data alone (e.g. common application of cytochrome c oxidase I in environmental DNA surveys) given the potential to conflate the two species, a limitation that can be overcome with nuclear markers^39^.

We also found evidence for hybridization and introgression of nuclear alleles in at least three *B. saida* individuals, two sampled in the Coronation Gulf of the Kitikmeot Region of Nunavut (Western Arctic ecoregion), and the third sampled between Prince of Wales Island and the southern tip of Somerset Island within the Eastern Arctic ecoregion. Previous work by Wilson et al.^32^ similarly identified a potential late generation hybrid with *A. glacialis* mtDNA and *B. saida* nuclear genotype sampled close to the Mackenzie River Delta, Beaufort Sea (Western Arctic ecoregion). The authors also simulated assignment probabilities following hybridization, and showed admixture proportions are practically negligible after more than 5 generations of backcrossing. Our proportions detected using maximum-likelihood ancestry models (i.e. ADMIXTURE; ca. 11/89%) were larger than those in the analysis of Wilson et al.^32^, suggesting hybridization between the two species is recent in Canada’s Western and Eastern Arctic ecoregions, despite millions of years of evolution between the two species^42^. Moreover, introgression of nuclear genomic information appears to flow in both directions across species, as suggested by the smaller *B. saida* proportion in one hybrid individual, and the smaller *A. glacialis* proportions in the remaining two hybrid individuals (Fig. 1C). We also noted around 3% (17 out of 511) of specimens collected as *B. saida* featured the mitochondrial DNA of *A. glacialis* (Table 1). In contrast, Nelson et al.^25^ removed a greater proportion of specimens with *A. glacialis* mitochondrial DNA (12%; 300 out of 2587), while Wilson et al.^32^ sampled 115 *B. saida* and 12 *A. glacialis* from Northern Alaska and the Western Canadian Arctic, with no mixing of mitochondrial and nuclear signals. Multiple reasons could explain these differences in reported introgression of *A. glacialis* mitochondrial DNA into *B. saida*, such as taxonomic expertise, sample sizes, and the extent/study areas, all of which varied considerably among studies. We hypothesize that differences in introgression rates may vary across the entire distribution range of *B. saida* (Table 1); confirming this would necessitate a broader study using a similar genome-wide genotyping approach.

### Inverted and sex-linked genomic regions in *Boreogadussaida*

We detected several genomic regions that contributed to important clustering patterns in population analyses of *B. saida*. The first dubious clustering pattern we detected on PC axes 3 and 4 of our initial population level PCA (Fig. 2A) was partially driven by two ca. 4–6 Mbp regions on chromosome 9 (Fig. 2C). Allele loadings (i.e. contributions to genomic signal) on axis 5 also pointed to a differentiated region on chromosome 5, and our downstream analyses pointed to a differentiated region on chromosome 14 (Fig. 4B,D). Given these regions were differentiated, with sites in high linkage disequilibrium (i.e. inherited as a unit; Fig. 2F), without any obvious spatial structure, and featured obvious clustering corresponding to high and low heterozygosity genotypes (Fig. S2), we concluded these regions represent large (i.e. Mbp) inverted regions of the genome. Large chromosomal inversions reduce recombination due to disruption of crossing over events in heterozygotes^43^, leading to regions of the genome with elevated linkage disequilibrium and FST (as observed here; Fig. 2F), and notable departures in heterozygosity between individuals lacking an inverted region (putatively ancestral AA and recently derived BB genotypes) and hosting one (AB; Fig. S2). We hypothesize the BB genotype represents two copies of the inverted region, given higher levels of homozygosity are expected when the large accumulation of alleles is brought back together. On chromosome 9, given two notable peaks, we speculate the ancestral state was a single inverted segment that broke into smaller inverted regions with varying levels of recombination (Fig. 2C,F, S2). Alternatively, these inverted regions were potentially established together at the same time. Chromosomal inversions have been reported in Atlantic cod (*Gadus morhua*), with similar PCA patterns in Northwest Atlantic populations reported by Puncher et al.^44^, and adaptive evolution inferred from these regions first reported by Bradbury et al.^45^. Sodeland et al.^34^ also link these “islands of divergence” to adaptive evolution in Atlantic cod, and hypothesize linkage groups are tailored to coastal and oceanic environments. Berg et al.^35^ also link these inversions to adaptive divergence in Northeast Arctic, Norwegian Coastal, and North Sea cod ecotypes. In Atlantic herring, a large inversion on chromosome 12 is associated with ecological adaptation to water temperatures and gonadal development, spawning, or early larval development^46^, while in capelin large chromosomal inversion(s) are also reported but not yet linked to ecological traits^47^. For *B. saida* in Canadian Arctic and subarctic waters, we found no obvious geographic specificity or ecological adaptations associated with the occurrence of the inverted repeat regions (Fig. S2). Nonetheless, chromosomal inversions suppress recombination in particular regions of the genome, creating the conditions for adaptive divergence in species with high gene flow, as appears to be the case in *B. saida*.

Inversions were reported for *B. saida* on chromosomes 1 and 7 by Einarrson et al.^36^, which are possibly derived from a common ancestor with Atlantic cod, or an introgression event. Curiously, however, we did not observe any evidence for divergent chromosomal segments corresponding to the inverted regions reported by Einarrson et al.^36^, including genomic regions in linkage disequilibrium on chromosomes 1 and 7 (Fig. 2F). Our results therefore indicate the inversions on chromosomes 1 and 7 are absent from Canadian populations, either because the inversion was lost or fixed, or because the inversions are potentially exclusive to Greenlandic populations (the study area of Einarsson et al.^36^). A larger scale analysis incorporating specimens from the entire circumpolar distribution range of *B. saida* is needed to determine the pervasiveness of these inversions. Einarsson et al.^36^ did not report on the putative inverted regions reported here from chromosomes 5, 9, and 14, and these regions were not previously reported to house chromosomal inversions in *G. morhua*. Our results therefore suggest *B. saida* chromosomal inversions have independently evolved, and are spatially segregated across its circumpolar distribution.

We also confirmed a region on chromosome 13 was linked to sex biased divergence (Fig. 2E). Determining factors of sexual differentiation in fish are highly variable, ranging from genetic sex determination with distinct chromosomes to diffuse loci, to determination through environmental conditions^48^. Here, we show that sex determination in *B. saida* is at least partially genetically driven through an XY mating system, though other factors are likely playing an important role given males and females did not cluster in our PCAs. In Atlantic cod, sex determining loci are found across five linkage groups, one of which closely approximated an XY mating system^49^. None of the linkage groups reported by Star et al.^49^, however, correspond to the region identified here, suggesting a rapid turnover of sexual determination in *B saida* or *G. morhua*, a phenomenon frequently reported in fish^50–52^.

Aside from nucleotide differences, we did not note any noteworthy functional differences specific to the inversion and sex-linked genomic clusters (e.g. Fig. S3). Future work would need to document or include information on habitat use/association, the occurrence of ontogenetic migrations, and prevailing environmental conditions (e.g. near-shore vs open ocean, brackish vs marine), as well as biological information (e.g. specimen size and timing of reproduction), to potentially unravel environmental effects and/or behavioral, morphological, or life history trait differences indicative of adaptation. The inverted regions (chromosomes 5 and 9) were associated with a wide variety of Gene Ontology terms, ranging from heme binding to cytoskeletal motor activity, while the sex-linked cluster contained genes associated with ion channel activity and metalloendopeptidase inhibition, though none of these regions were significantly enriched with particular functions (Fig. S3). Whether these genomic regions are of any adaptive significance requires further analysis, ideally leveraging a reference genome for *B. saida* rather than *G. morhua*. Note also, given the linkage disequilibrium results (Fig. 3F), more inversions could be confirmed in future analyses.

### Population structure of Canadian *B. saida* reflects regional scale structuring and potential adaptive evolution

Our analysis of population structure in *B. saida* revealed clear spatial patterns across the species range within Canada (Fig. 3A), with notable structure broadly corresponding to three ecoregions/areas: (1) the Western Arctic, (2) the Eastern Arctic, and (3) the area comprising the Hudson Bay Complex, the Newfoundland-Labrador Shelves, and the Gulf of St. Lawrence ecoregions (Fig. 3D–F). This structure supports the findings of Nelson et al.^25^, wherein microsatellites reflected similar population differentiation in the Eastern versus Western North American Arctic. Our study moreover confirmed that some specimens from the Hudson Bay Complex through to subarctic waters of the Newfoundland and Labrador shelves and Gulf of St. Lawrence ecoregions are also differentiated from higher Arctic locations (Fig. 3). Some population structure (i.e. Q4, Fig. 3G) was especially associated with the Newfoundland-Labrador shelves, where heterozygosity was also notably high (Fig. 3C). Several hypotheses might explain these patterns. The Arctic environment was recently covered with thick-ice sheets during the last glacial maximum^41^, which presumably extirpated the highly productive under ice algae and zooplankton that sustain oceanic Arctic ecosystems^6^. As such, *B. saida* populations were likely also extirpated from the Arctic during glaciations, surviving in glacial refugia isolated in the North Pacific and North Atlantic oceans, as inferred in widespread marine lineages including various invertebrates^53^, sea stars^54^, polychaetes^55^, urchins^56^, and seaweeds^57–59^. Isolation into separate Atlantic and Pacific refugia could explain the divergence between Western Arctic and Newfoundland and Labrador Shelves ecoregions (Fig. 3B). The Eastern Arctic, meanwhile, could be the result of recent range expansions, either from Atlantic or Pacific refugia. The overall low FST values (Fig. 3B), weak structure globally^16–23,25^, and high migration potential, does not exclude the possibility of circumpolar expansion from a single basin following glaciation, followed by differentiation of globally established populations, or some combination of the above scenarios. Increased geographic sampling of populations and demographic modelling are needed to unravel the series of events and timeline for evolution in *B. saida* populations.

The high heterozygosity of the subarctic Canadian population (Hudson Bay Complex, Newfoundland and Labrador Shelves and Gulf of St. Lawrence), however, does not support the hypothesis of recent Arctic recolonization from a single source population, as these results suggest the subarctic population is outbred. Glaciation is also known to have pushed marine populations into East vs West Atlantic refugia^60–62^, and the work of Nelson et al.^25^ indicated European Arctic populations are differentiated from Canadian populations. Bigg et al.^63^ also support the view of refugia scattered across the Atlantic during the last glacial maximum for *G. morhua*, with solid agreement between paleoecological models and a variety of genetic data. The signal unique to *B. saida* inhabiting subarctic waters of Canada’s East coast, combined with the observation of high heterozygosity in some individuals, points to potential interbreeding with another differentiated population. One possibility is interbreeding between DAPC clusters 2 and 3 (Fig. 3A), which is somewhat supported by high heterozygosity at the facing margins of clusters (Fig. S4). Refugial Greenlandic populations are also worth considering, a hypothesis supported in kelp populations both through ecological niche modelling^64^, and genomic datasets^65^. A global scale analysis of genome-wide SNPs is needed to determine the origin of the unique population signal and high heterozygosity encountered in specimens from the three subarctic ecoregions.

We also observed that the population structure reported here was excessively driven by a small number of loci, largely aggregated in 13.8% of the SNPs and into particular genomic regions scattered across chromosomes, a finding also reflected in the linkage disequilibrium results (Fig. 4A). Though the reported population structure remains in the neutral loci (Fig. 4E,F, S6), the observation that population structure is disproportionately driven by a relatively small number of loci suggests adaptive evolution is playing an important role in globally distributed *B. saida* populations^66^. One possibility is that these adaptive regions may also represent smaller chromosomal inversions, meaning many, potentially more recent, inversions are playing a role in the evolution of *B. saida* populations. Examples of adaptive evolution located in inversions are suggested in Atlantic cod^45,67^, and islands of adaptive divergence facilitated by inversions are reported in freshwater and marine threespine sticklebacks (*Gasterosteus aculeatus*^68^). In the case of *B. saida*, divergence has been reported between fjord-dwelling and shelf-dwelling populations in the East Atlantic^23^, but this mechanism for adaptive evolution, among others explored here, is supported with little direct evidence (though see Hill^24^). A hypothesis worth exploring is whether specimens from the subarctic ecoregions (Hudson Bay Complex, Newfoundland and Labrador Shelves and Gulf of St. Lawrence) are adapted to a shorter ice season and thus a more pelagic habitat. A more conservative, less adaptation oriented conclusion of our results, is that genetic drift plays a minor role in structuring populations given there is little random structure present in Canadian populations. Explanations for this include consistently large effective population sizes possibly mediated by complex source-sink dynamics or recently expanded populations (see above discussion on Arctic recolonization; see also positive impact of expansion on false discovery rate of outlier SNPs in Luu et al.^69^). Of course, a dominant role of adaptive evolution^45,66–68^, or a combination of neutral and non-neutral factors are viable possibilities. An analysis of functional differentiation of populations would benefit immensely from a reference genome for *B. saida*, along with whole genome sequence information to pinpoint peaks in adaptive signal^70–72^, and environmental and biometric/life history data that could be coupled with an analysis of functional variation among specimens.

### Conclusions

*Boreogadus saida* is a key, mid-trophic level species modulating energy flows between lower and higher levels in Arctic ecosystems. Our genome wide analysis of population SNPs provides important context for understanding the potential vulnerability of this species to ongoing climate change and direct anthropogenic stressors. In particular, if West vs East Arctic and subarctic regions are functionally differentiated, we could expect different responses to climate change across the Canadian range. The introgression between *B. saida* and *A. glacialis* could also have functional implications in the future, particularly in the Western Arctic where hybridization occurs. If populations are not yet functionally differentiated, ample substrate exists for selection given three putative chromosomal inversions (chromosomes 5, 9, and 14) and the linked outlier loci across chromosomes in Canadian *B. saida* populations. A reference genome for *B. saida*, along with sampling from the entire circumpolar species distribution range, corroborated with environmental and biometric/life history data, are needed to confirm the positions of SNPs and inversions and to delve further into the functional relevance of the patterns observed here. Doing so will provide further genomic context for the protection of the species and ecosystems that depend on *B. saida*.

## Methods

### Data collection, DNA extraction, and sequencing

Individuals were collected with bottom or pelagic trawls during scientific surveys (CCGS *Amundsen*, CCGS *Teleost*, CCGS *Leim*, the Aqviq and the Katsheshuk II of the Northern Shrimp Research Foundation—Fisheries and Oceans Canada) in five marine ecoregions (i.e., Canadian Marine Areas^73^): the Western Arctic (2021, n = 123) and the Eastern Arctic (2021, n = 175), the Hudson Bay Complex (2005, 2021, n = 63), the Newfoundland and Labrador Shelf (2021, n = 149) and the Gulf of St. Lawrence (2020, 2022, n = 66; Fig. 1A; Table S1). A few individuals were also sampled from ice fishing in the Saguenay Fjord (2019, n = 26), considered here as the Gulf of St. Lawrence ecoregion. The ecoregions used for analyses are high-level spatial units primarily based on oceanographic and bathymetric similarities^73^. Individuals were measured and sex was determined visually. Muscle tissue or fin clips were preserved in EtOH 95% prior to the DNA extraction.

DNA was extracted with DNeasy Blood and Tissue Kits (Qiagen). Libraries of ddRAD were prepared by the Plateforme d’analyse génomique (IBIS, Université Laval) using *PstI* and *MstI* restriction enzymes and 20 ng of DNA per sample [see detailed protocol in^74^. Six libraries of 96 samples (570 individuals and 6 duplicates) were sequenced on one lane of NovaSeq 6000 S4 PE150 (NovaSeq 6000 S4 Reagent Kit v1.5, 300 cycles) by Génome Québec (Montréal, Canada) with 10% PhiX. The targeted sequencing depth was > 10x.

### Read processing, SNP calling and filtering

Illumina adaptors were first removed using Trimmomatic v0.39^75^. Then, demultiplexing and quality filtering were performed with the *process_radtag* module of Stacks v2.64^76,77^ with a truncation at 90 bp and check for restriction cut sites. Given the lack of reference genome in *B. saida* and previous work in *B. saida* mapping to the reference genome of Atlantic cod^36^, reads were aligned to the chromosomal level reference assembly GadMor3 (GCF_902167405) with *mem* from BWA-MEM^78^ and default parameters. Ten samples with low numbers of mapped reads (< 10 millions) were discarded.

The *gstacks* module was used to create a SNP catalog, then the *population* module was run over high quality samples (mean coverage > 10x) to compile into a Variant Call Format (VCF) file. SNPs with a minor allele frequency greater than 1%, and present in over 75% of samples were kept in the dataset, while one sample with > 30% missing values was removed from the dataset. Next, SNPs with > 10% missing values, with expected heterozygosity > 0.6, and with low (< 10x) and high (> 95x) median coverage were removed with VCFtools^79^. Relatedness coefficients were checked using the relatedness2 function of VCFtools and one individual each from two pairs of related individuals (Φ < 0.3) were removed. Finally, to avoid SNPs in linkage disequilibrium (i.e. in close proximity on the genome), only the first SNP of each ddRAD loci was kept (Table 2).

**Table 2:** Counts of ddRADseq single nucleotide polymorphisms (SNPs), loci, and samples after filtering steps. SNPs/loci are carried forward at each step to progressively filter data, thus, each row includes filtering steps above. Filtering steps indicate criteria to retain SNPs, loci or samples.

Filtration step	SNPs	loci	N
Initial samples			576
Mapped reads > 10 millions			566
SNPs catalog creation (gstacks)		1,046,093	566
Minimum 10 × coverage		1,046,093	532
Minor Allele Frequency (MAF) > 1% and SNP detected in at least 75% of individuals	227,266	84,220	532
Individuals with < 30% missing SNPs, and SNPs with < 10% missing values	191,001	56,312	526
Observed heterozygosity lower than 60%	190,049	56,049	526
SNPs with coverage between 10 and 105x (approx.1–99% percentile)	184,620	53,550	526
Relatedness (Φ) < 0.3	184,620	53,550	522
Species level analyses			
1 SNP per locus, MAF > 1%	53,384	53,384	522
Analyses of inversions and sex-biased loci			
Minor allele frequency > 5% and *Arctogadus glacialis* and hybrid individuals removed	12,305	12,305	511
Sites with elevated axis/allele loadings on chromosome 5 removed (i.e. putative inversion)	11,991	11,991	511
Sites with elevated axis/allele loadings on chromosome 9 removed (i.e. putative inversion)	11,428	11,428	511
Sex linked loci on chromosome 13 removed	11,233	11,233	511
Analyses of outlier SNPs			
Outlier loci removed based on PCAdapt q-values	9,680	9,680	511
Sites with elevated axis/allele loadings on chromosome 14 removed (i.e. putative inversion)	9,458	9,458	511
Analyses of neutral population structure			
One SNP per linkage group (r^2^ > 0.05 within 10 Mbp; r^2^ > 0.10 inter-chromosome) retained	7,133	7,133	511

### Genomic analyses

SNPs located within the mitochondrial genome (i.e. haplotpes) were explored to assess mitochondrial identity, particularly for potential misidentification with *A. glacialis*. Raw reads were first aligned to the *B. saida* reference mitogenome (NC_010121.1). Then, haplotypes starting in three regions with high density observations (position 4894, 5138, 9134) were blasted to a local version of NCBI-nt (downloaded 2022-01-04) to assign them a taxonomic identity (Top hit approach, 95% identity threshold). Five individuals were targeted for the sequencing of the Folmer region of cytochrome C oxidase using the LCO1490 and HCO2198 primers^80^ to confirm which haplotypes were discriminants. We determined that one haplotype was discriminant for *B. saida* (position 5138) while another was discriminant for *A. glacialis* (position 4894). These two haplotypes were used to genotype mitogenomes of all specimens (Table S2).

Nuclear SNP information was also used to confirm species identity and remove *A. glacialis* and potential hybrids prior to population analyses of *B. saida*. Principal component analyses (PCAs) were conducted on the full dataset (522 individuals, 53,384 SNPs) using the *glPca* function of *adegenet* v.2.1.10^81,82^ in R^83^, with alleles centered and missing values converted to the genotype mean across all individuals. Ancestral proportions were estimated with fivefold cross-validation using ADMIXTURE v.1.3.0^84^. Individuals with nuclear DNA corresponding to *A. glacialis* or potential hybrids were removed using VCFtools v.0.1.13, leaving 12,305 SNPs for analysis (also removing SNPs with a minor allele frequency < 0.05; Table 2).

In order to detect biased regions of the genome, PCA was performed on the data subset consisting of only *B. saida* individuals (n = 511 individuals and 12,305 loci). Several clusters lacking geographic specific associations were noted, and allele loadings were inspected for patterns indicative of inversions (i.e. loadings aggregated to a specific region). Demultiplexed reads were also used to identify genomic regions associated with the sex of individuals. RADSex^85^ was used to sort unaligned raw reads and determine their presence/absence in each individual, allowing the identification of sex-biased reads. We used forward (R1) reads as input for RADSex along with sex identity of 255 individuals (144 females, 111 males) to identify reads significantly biased toward one sex (min depth = 5x, with Bonferroni correction). The sorted reads were also mapped to the Atlantic cod reference genome (GadMor3; GCF_902167405) to identify the genomic region(s) of sex-biased reads. These patterns were further confirmed by screening genomic regions for linkage disequilibrium, calculated using plink v1.9^86^. For linkage disequilibrium, r^2^ values were averaged at the site level, pairing sites at a distance of 100 kbp–5 Mbp.

Inversions and sex-linked regions were removed from the dataset (chromosome 5/LR633947.1, positions 3,622,906–13,963,306; chromosome 9/LR633951.1, positions 7,350,621–11,315,580 and 17,597,431–23,417,670; chromosome 13/LR633955.1, positions 11,053,431–27,975,038), leaving 11,233 SNPs. Functions of the removed regions were investigated using Ensembl gene codes and Gene Ontology (GO) terms^87,88^. First, the above biased gene regions and corresponding annotations were extracted from the GadMor3 reference genome (GCF_902167405.1) using NCBI’s genome data viewer and gene search engine. Enrichment analyses were performed using Ensembl gene identifiers and the *G. morhua* organism option and g:SCS threshold correction for multiple testing in g:Profiler^89^. Molecular function GO terms from the g:Profiler output were then reduced using Multidimensional Scaling using the default SimRel similarity measure in REVIGO v.1.8.1 (REduce + VIsualize Gene Ontology^90^). The scatter plot output was further edited in R.

In order to interpret population structure in *B. saida*, PCA, DAPC, and ADMIXTURE were performed on the subsequent dataset with inversions and sex-linked regions removed (n = 11,233 loci). PCA was performed as above using the *glPca* function of *adegenet*, while ADMIXTURE was performed from *k* = 1–10. For DAPC, the find.clusters function of *adegenet* was used to define groups, retaining 400 PCs (80% of variation) and three clusters based on Bayesian Information Criterion (Fig. S1A,B). Allele loadings for the discriminant functions were then inspected on axes 1 and 2.

Further suspect clustering patterns were noted, so SNPs were screened for potential outliers, i.e., SNPs explaining larger than expected proportions of population structure. We first used PCAdapt v4.3.5^91^ and an individual based-method to identify outliers SNPs in the two first PC axes. We also used Bayescan v.2.1^92^ to identify SNPs with higher than expected FST values among the five marine ecoregions. For both approaches, SNPs with a *q*-value < 0.05 were identified as outliers, and outliers based on Bonferroni correction were also explored in the PCAdapt results. Plink was also used to remove linked sites with an r^2^ value > 0.05 and within 10 Mbp, and with an r^2^ value > 0.1 across chromosomes. A final PCA was performed as above on the resulting no-hybrids, no-inversions, no-sex-linked segment, and no-outlier dataset (n = 7133 loci), which was used to interpret population structure in *B. saida.*

Heterozygosity was calculated for each individual using the –het function in VCFtools. Pairwise ecoregion FST was estimated using the *gl.fst.pop* function of *dartR*^93,94^, using 1000 bootstrap replicates to test for significant departures from 0. Kruskal–Wallis tests were used to test for significant differences in heterozygosity among ecoregions using the *stats* R package, followed by Dunn’s post hoc tests to determine what ecoregions, if any, were significantly different from each other using the *dunn.test* R package^95^. Plots were constructed using *ggplot2*^96^. Length and weight measurements were tested for significant differences across regions using the *agricolae* package in R^97^. Departures across regions were tested using Tukey’s Post Hoc test.

### Supplementary Information

Supplementary Information 1.

Supplementary Table S1.

## Data Availability

All sequence data can be accessed via NCBI’s BioProject PRJNA1062734. Workflow commands are available via Github: https://github.com/GenomicsMLI-DFO/Boreogadus_ddRADseq_2022.
