# Supplementary material for: Genomic architecture and population structure of Boreogadus saida from Canadian waters
Source: Sci Rep. 2024 Aug 20;14:19331. doi: 10.1038/s41598-024-69782-w (PMC11336163; doi:10.1038/s41598-024-69782-w)
Supplement: Supplementary file 1 — Supplementary Information 1. [file 41598_2024_69782_MOESM1_ESM.docx]

**Supplemental material**

**Table S1.** Specimen information for *Boreogadus saida*. Provided online.

**Table S2.** Detailed results on classification of mtDNA identity based on two mitogenome haplotypes. Those with an asterisk (*) were confirmed by sequencing cytochrome c oxidase Folmer sequence (5 individuals).

| **mtDNA identity** | **Taxonomy assigned to two discriminant SNPs** | **N** |
| --- | --- | --- |
| *A. glacialis* | *A. glacialis* / Gadidae* | 22 |
|  | *A. glacialis* / Gadiformes | 4 |
|  | *A. glacialis* / NA | 7 |
| *B. saida* | *B. saida* / *B. saida* | 41 |
|  | NA / *B. saida** | 283 |
| Undefined | *A. glacialis* / *B. saida* | 26 |
|  | *B. saida* / Gadidae | 5 |
|  | *B. saida* / Gadiformes | 3 |
|  | *B. saida* / NA | 2 |
|  | *Gadus morhua* / Gadiformes | 1 |
|  | NA / Gadidae | 51 |
|  | NA / Gadiformes | 10 |
|  | NA / NA | 121 |

**
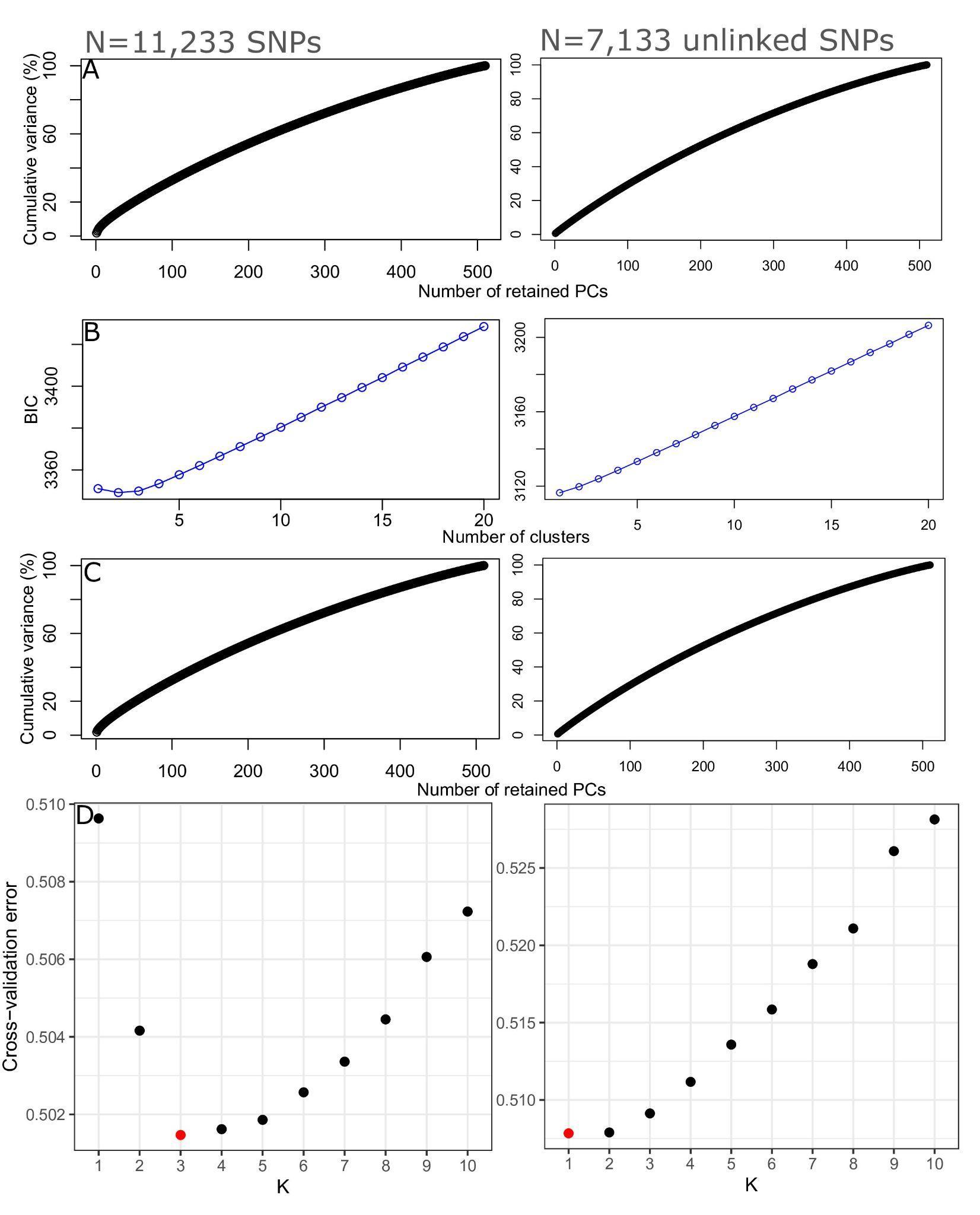

Figure S1.** Cluster analyses in Canadian *Boreogadus saida*. A) Variation explained by PC axes using the find.clusters function in *adegenet* v.2.1.10 for linked and unlinked loci, respectively. B) BIC for number of clusters based on 400 axes retained for linked and unlinked loci, respectively. C) Variation explained by PC axes using the dapc function in *adegenet* v.2.1.10 and using the groups identified using find.clusters (400 axes retained), for linked and unlinked loci, respectively. D) Cross-validation errors for different levels of *k* based on ADMIXTURE analyses, for linked and unlinked loci, respectively.


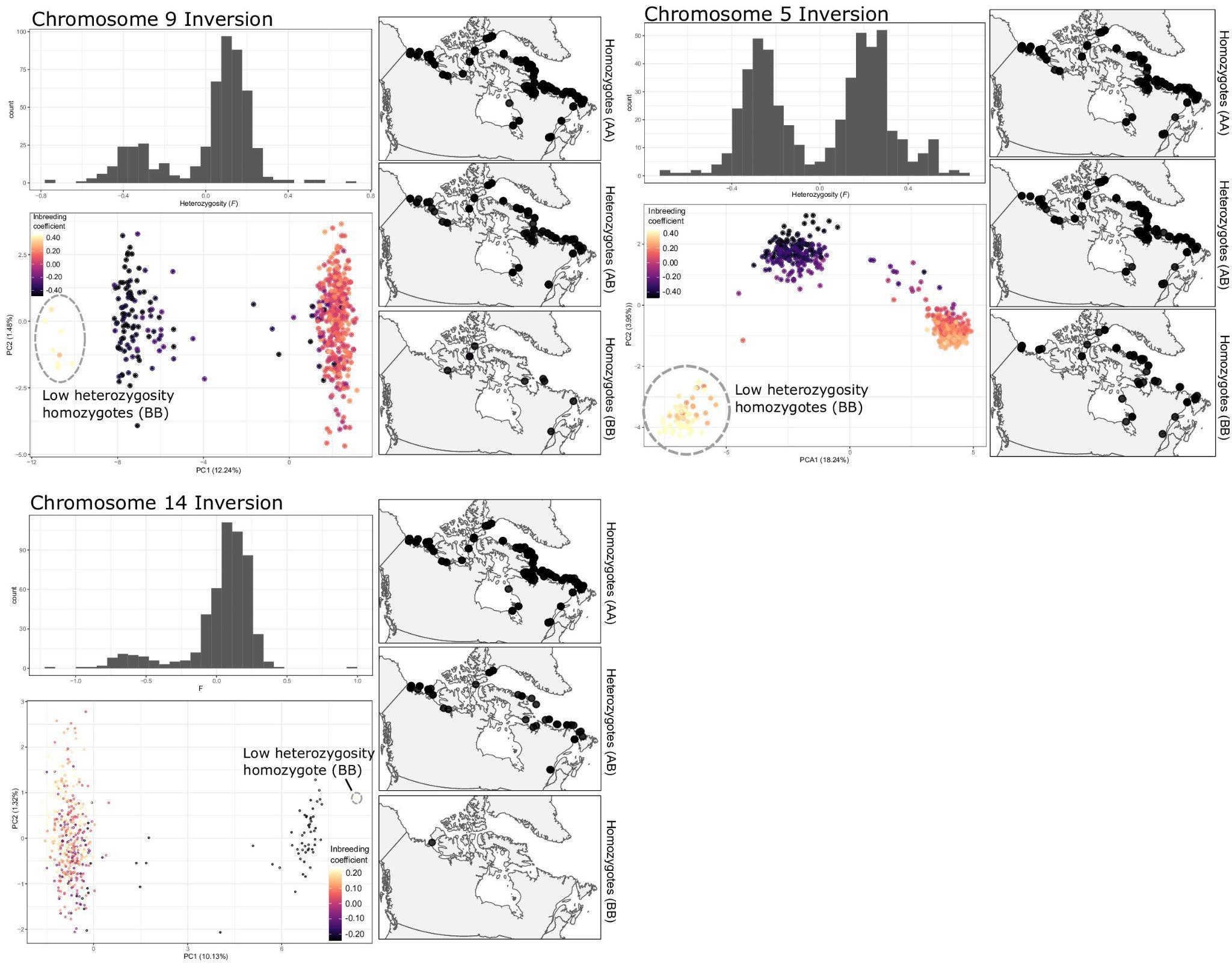


**Figure S2.** Distribution of inbreeding coefficients (*F*) in homo/heterozygotes for chromosomes 5, 9, and 14 inversion in Canadian *Boreogadus saida*.

**
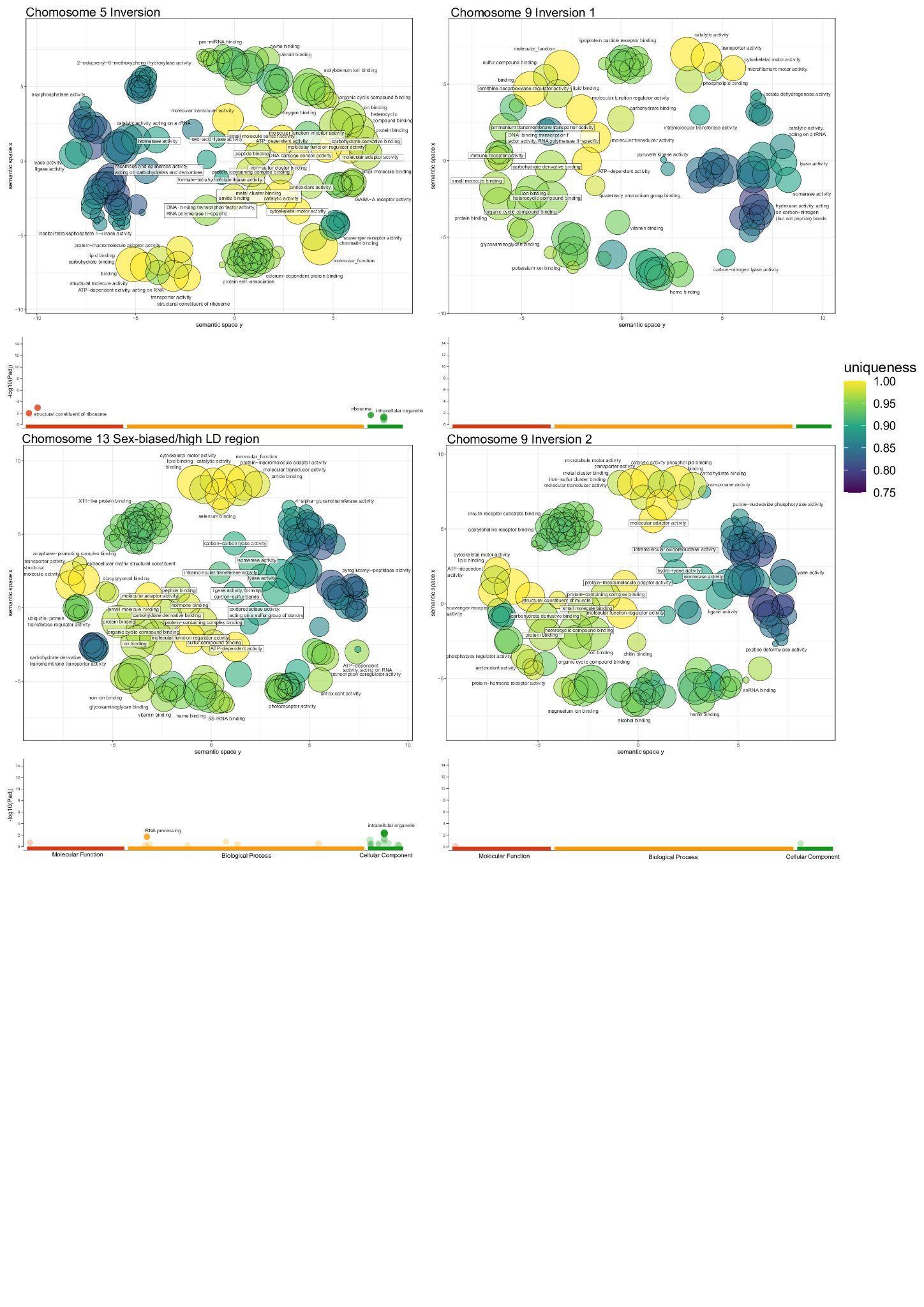
**

**Figure S3.** Gene ontology results for inversions on chromosomes 5 and 9, and a sex-biased region on chromosome 13 in Canadian *Boreogadus saida*. Gene Ontology (GO) terms are for molecular functions in the biased regions, reduced using multidimensional scaling based on pairwise semantic similarities. Uniqueness is calculated as 1-(average semantic similarity to all other terms); the size of the circles proxies the generality of terms, calculated as Log10 (number of annotations for GO term in the underlying GOA database).

**
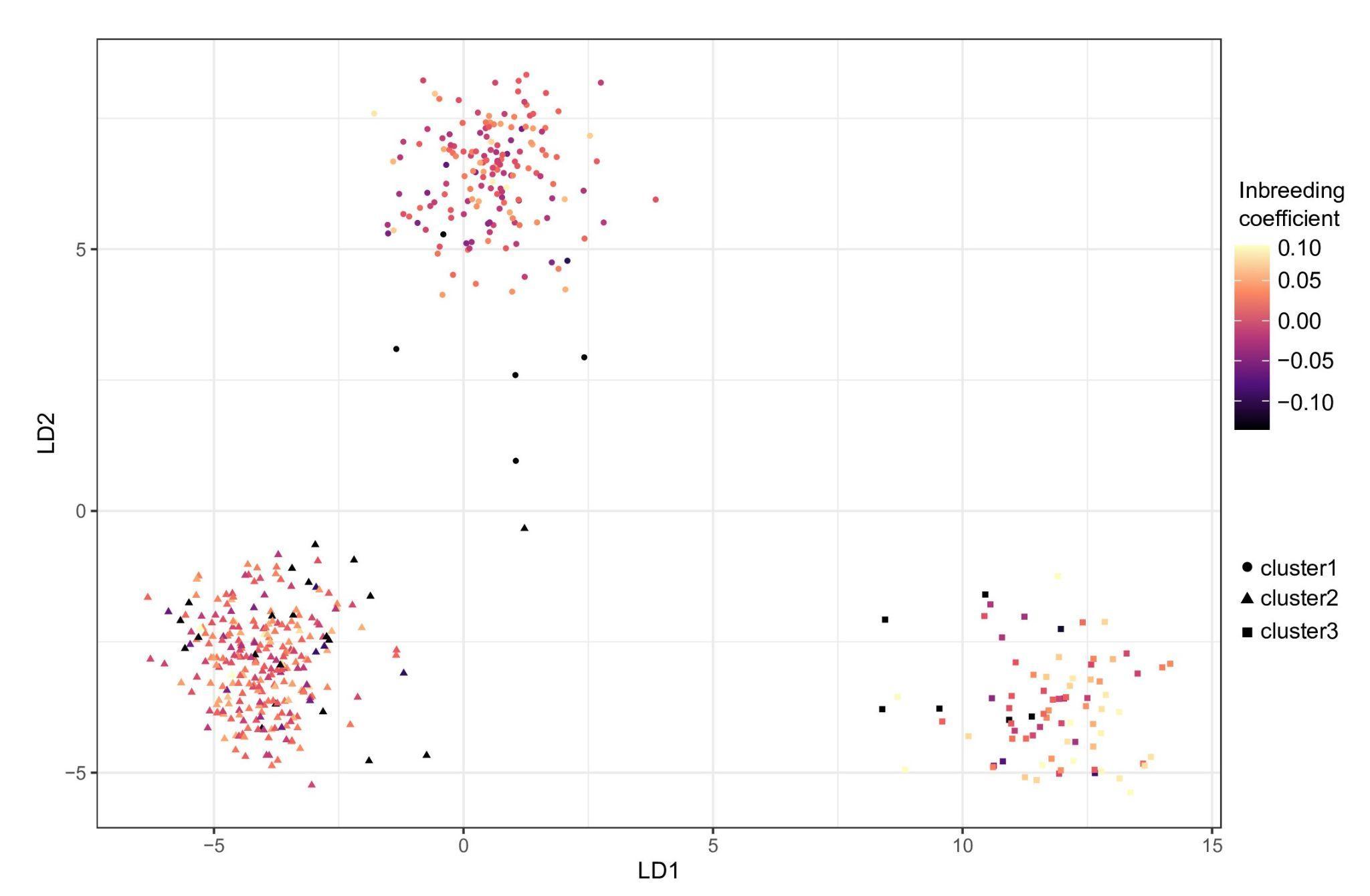
Figure S4.** Discriminant analysis of principal components (DAPC) results colour coded for inbreeding coefficients (*F*) in Canadian *Boreogadus saida* (corresponds to Fig. 3A).


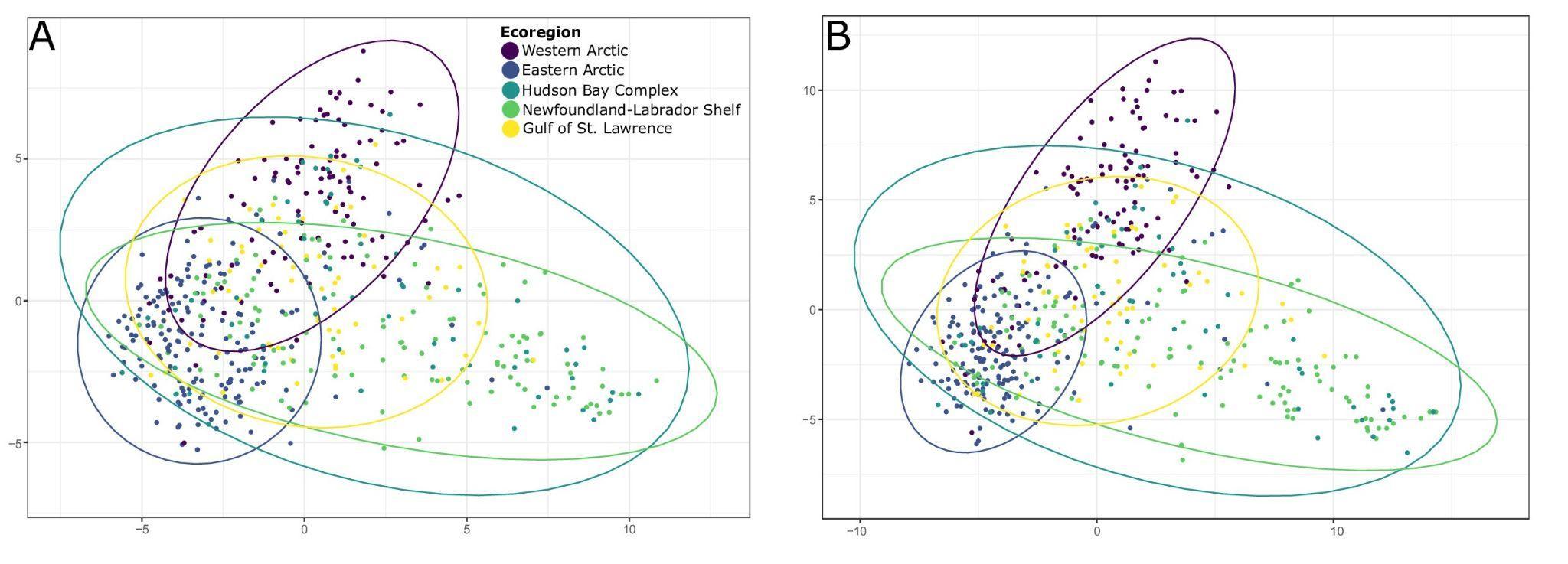


**Figure S5.** PCA results for outlier loci determined using PCAdapt as defined by A) Bonferroni correction and B) q-values in Canadian *Boreogadus saida*.


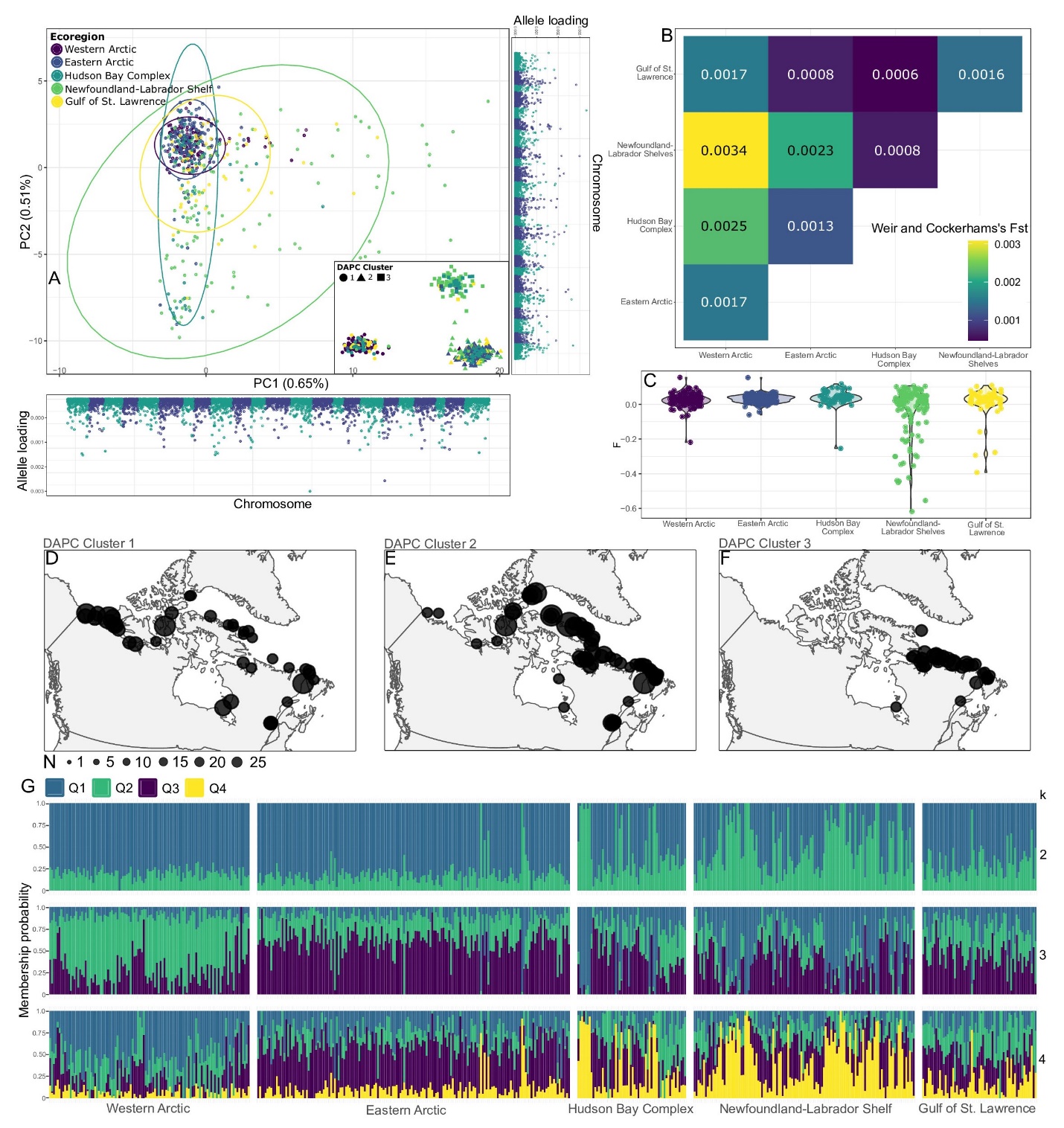


### Figure S6. Population structure of Canadian *Boreogadus saida* using unlinked loci (n=7,133 single nucleotide polymorphisms, SNPs). Population results include: A) Principal component analysis with Discriminant Analysis of Principal Component (DAPC) inset and discriminant analysis allele loading profiles depicted for each axis (assuming 3 clusters and ca. 80% of variation retained during analysis); B) Weir and Cockerham’s Fst pairwise measurements for each population (all pairwise estimations were significantly different from 0); C) Inbreeding coefficients; geographic distribution of DAPC clusters 1-3 (D-F, respectively), where N refers to number of individuals genotyped; and G) ADMIXTURE results at k=2-4 (k=1 was best supported based on cross-validation error (Fig. S1).
